# Comparison of four commercial, automated antigen tests to detect SARS-CoV-2 variants of concern

**DOI:** 10.1007/s00430-021-00719-0

**Published:** 2021-08-20

**Authors:** Andreas Osterman, Maximilian Iglhaut, Andreas Lehner, Patricia Späth, Marcel Stern, Hanna Autenrieth, Maximilian Muenchhoff, Alexander Graf, Stefan Krebs, Helmut Blum, Armin Baiker, Natascha Grzimek-Koschewa, Ulrike Protzer, Lars Kaderali, Hanna-Mari Baldauf, Oliver T. Keppler

**Affiliations:** 1 Max Von Pettenkofer Institute and Gene Center, Virology, National Reference Center for Retroviruses, LMU München, Munich, Germany; 2 German Center for Infection Research (DZIF), Partner Site, Munich, Germany; 3 COVID-19 Registry of the LMU Munich (CORKUM), University Hospital, LMU Munich, Munich, Germany; 4 Laboratory for Functional Genome Analysis, Gene Center, LMU München, Munich, Germany; 5 Public Health Microbiology Unit, Bavarian Health and Food Safety Authority, Oberschleißheim, Germany; 6 Institute of Virology, Technical University of Munich/Helmholtz Zentrum München, Munich, Germany; 7 Institute of Bioinformatics, University Medicine Greifswald, Greifswald, Germany; 8 Max Von Pettenkofer Institute, Virology, National Reference Center for Retroviruses, LMU München, Feodor-Lynen-Str. 23, 81377 Munich, Germany; 9 Max Von Pettenkofer Institute, Virology, National Reference Center for Retroviruses, LMU München, Pettenkoferstr. 9a, 80336 Munich, Germany

**Keywords:** Automated SARS-CoV-2 antigen test, Nucleocapsid protein, Diagnostic test, Sensitivity, Specificity, VOC

## Abstract

A versatile portfolio of diagnostic tests is essential for the containment of the severe acute respiratory syndrome coronavirus type 2 (SARS-CoV-2) pandemic. Besides nucleic acid-based test systems and point-of-care (POCT) antigen (Ag) tests, quantitative, laboratory-based nucleocapsid Ag tests for SARS-CoV-2 have recently been launched. Here, we evaluated four commercial Ag tests on automated platforms and one POCT to detect SARS-CoV-2. We evaluated PCR-positive (*n* = 107) and PCR-negative (*n* = 303) respiratory swabs from asymptomatic and symptomatic patients at the end of the second pandemic wave in Germany (February–March 2021) as well as clinical isolates EU1 (B.1.117), variant of concern (VOC) Alpha (B.1.1.7) or Beta (B.1.351), which had been expanded in a biosafety level 3 laboratory. The specificities of automated SARS-CoV-2 Ag tests ranged between 97.0 and 99.7% (Lumipulse G SARS-CoV-2 Ag (Fujirebio): 97.03%, Elecsys SARS-CoV-2 Ag (Roche Diagnostics): 97.69%; LIAISON^®^ SARS-CoV-2 Ag (Diasorin) and SARS-CoV-2 Ag ELISA (Euroimmun): 99.67%). In this study cohort of hospitalized patients, the clinical sensitivities of tests were low, ranging from 17.76 to 52.34%, and analytical sensitivities ranged from 420,000 to 25,000,000 Geq/ml. In comparison, the detection limit of the Roche Rapid Ag Test (RAT) was 9,300,000 Geq/ml, detecting 23.58% of respiratory samples. Receiver-operating-characteristics (ROCs) and Youden’s index analyses were performed to further characterize the assays’ overall performance and determine optimal assay cutoffs for sensitivity and specificity. VOCs carrying up to four amino acid mutations in nucleocapsid were detected by all five assays with characteristics comparable to non-VOCs. In summary, automated, quantitative SARS-CoV-2 Ag tests show variable performance and are not necessarily superior to a standard POCT. The efficacy of any alternative testing strategies to complement nucleic acid-based assays must be carefully evaluated by independent laboratories prior to widespread implementation.

## Introduction

Non-PCR-based point-of-care testing (POCT) has been widely introduced into national test strategies and independently evaluated using different settings and approaches [1–4]. While the detection and quantification of SARS-CoV-2 genomes by nucleic acid amplification testing represent the gold standard in diagnostic laboratories, the reagent supply chain can be limiting and turnaround times for PCR testing prolonged, making an effective clinical and outbreak management difficult at times, especially when incidences are high.

To add to the repertoire of quality-controlled, laboratory-based SARS-CoV-2 testing from respiratory material, several companies have recently introduced automated, quantitative SARS-CoV-2 Ag assays. First field studies indicate that these medium- to high-throughput assays’ sensitivities ranged from 40 to 93% and specificities between 91 and 100% [5–16]. While assay specificities were frequently found to be relatively high [5–16], assay sensitivities according to international and national guidelines for rapid Ag tests in general, requiring positive rate percentages ≥ 80, have frequently not been met [17, 18]. Further, the studies published so far did not compare different automated SARS-CoV-2 Ag assays among each other, which will be critical for diagnostic laboratories seeking to implement such assays.

Since the beginning of 2021, VOCs have started to dominate the pandemic in different parts of the world. For Germany, the Robert-Koch Institute estimated that in the middle of April 2021 ca. 90% of newly diagnosed SARS-CoV-2 infections were caused by VOC Alpha (pangolin lineage B.1.1.7), in line with surveillance reports from many other countries [19, 20]. In South Africa, the VOC Beta (pangolin lineage B1.351) has been driving the second wave in late 2020/early 2021 [21] and has been detected around the globe, including many European countries [19, 22]. It is largely unclear whether SARS-CoV-2 Ag assays are able to sensitively detect VOCs, which besides their defining mutations in spike also carry up to four amino acid mutations in the respective nucleocapsid proteins, i.e., Alpha: D3L, R203K, G204R, S235F; Beta: T205I; Gamma: P80R, R203K, G204R; Delta: D63G, R203M, G215C, D377Y [23].

The aim of our study was to compare four different automated SARS-CoV-2 Ag assays, which all detect the nucleoprotein of SARS-CoV-2 applying different technologies, for their analytical performance. In parallel, a widely used POCT rapid Ag test was used with an identical set of samples. We evaluated respiratory samples collected from patients at the University Hospital of Munich (LMU Klinikum) at the end of the second pandemic wave in Germany (February–March 2021) as well as patient-derived isolates, including EU1 (pangolin lineage B.1.177), VOC Alpha (B.1.1.7) and VOC Beta (B.1.351), which had been expanded in cell culture in a biosafety level 3 laboratory.

## Materials and methods

### Respiratory swabs

In the period February 1 to March 1, 2021, respiratory swabs (nasopharyngeal, oropharyngeal or unrecorded sampling site) were collected by health-care professionals from individuals, who were seen in the emergency room or on clinical units of the LMU Klinikum, the second-largest University Hospital in Germany, and three teaching hospitals of the LMU Munich (Helios Amper Hospital Dachau, Helios Hospital München West and Helios Hospital München Perlach). For this study, flocked swabs were collected in IMPROVIRAL™ with 3 ml Viral Preservative Medium (VPM) (Improve Medical, Guangzhou, Republic of China) or CITOSWAB^®^ with 3 ml Viral Transport Medium (VTM) (Citotest Scientific Co.,Ltd, Jiangsu, Republic of China) and analyzed by RT-PCR for SARS-CoV-2 RNA. All samples with a measurable Cp/Ct value by RT-qPCR under accredited conditions were scored “SARS-CoV-2-positive”. Analysis for the lower limit of detection was not performed in this study, but analyzed elsewhere [24]. Original respiratory swabs and transport media were stored at 4 °C for up to 24 h, until samples were inactivated and SARS-CoV-2 Ag testing was performed. A total of 107 SARS-CoV-2-PCR-positive and 303 PCR-negative respiratory samples were analyzed.

### SARS-CoV-2 antigen tests

All tests were performed according to the manufacturer's instructions. The swab set/transport media and specimen storage conditions described above do not deviate from the manufacturer's recommendations unless further specified below.

The SARS-CoV-2 Rapid Antigen Test (“RAT”) from Roche Diagnostics (Rotkreuz, Switzerland) is a rapid chromatographic immunoassay intended for the qualitative, visual detection of SARS-CoV-2 present in the human nasopharynx [25]. This assay is based on mouse monoclonal antibodies against SARS-CoV-2 nucleocapsid. Besides the extraction buffer provided, the manufacturer recommends the use of three specific VTMs. The presence of a test line (T), no matter how faint, together with a control line (C) means a positive test result. The manufacturer proposes it as a screening test in POCT settings for both symptomatic and asymptomatic individuals and states in the product sheet a test sensitivity of 96.52% and a test specificity of 99.68% based on results from studies conducted in Israel and Brazil referred to in the product sheet [25].

The Lumipulse G SARS-CoV-2 Ag (“CLEIA”) from Fujirebio Inc. (Tokyo, Japan) is an assay system containing a range of immunoassay reagents for the quantitative measurement of SARS-CoV-2 Ag in samples based on CLEIA technology [26], a specific two-step immunoassay on the LUMIPULSE G system [27]. This assay uses several monoclonal antibodies against SARS-CoV-2 nucleocapsid coated on ferrite particles. When preparing the sample from virus preservative solutions for nucleic acid testing, the manufacturer recommends centrifuging the sample at a minimum of 2000×*g* for at least 5 min and using the supernatant for measurement. Using a cutoff of 1.34 pg/ml, the sensitivity was 91.7% and the specificity 97.3% in a study of 325 specimens from Japanese hospitals (nasopharyngeal swabs with virus preservative solution) [27]. The manufacturer recommends the determination of an individual cutoff value according to the requirements of the respective laboratory.

The LIAISON^®^ SARS-CoV-2 Ag (“CLIA”) assay from DiaSorin S.p.A. (Saluggia, Italy) utilizes a direct, two-step sandwich chemiluminescence immunoassay (CLIA) for the quantitative determination of SARS-CoV-2 in nasal swabs or nasopharyngeal swabs [28]. The test uses rabbit polyclonal antibodies against the SARS-CoV-2 nucleocapsid. Furthermore, the manufacturer restricts use to individuals with suspected COVID-19 within 10 days of symptom onset. The test may only be performed on the LIAISON^®^ XL Analyzer.

For nasopharyngeal swabs, the clinical performance of the LIAISON^®^ SARS-CoV-2 Ag test was determined using a total of 408 samples from symptomatic patients. The sensitivity is reported to be 99.1% and the specificity 98.7%. The manufacturer specifies in the package insert that nasopharyngeal swabs in VTM/UTM should only be stored at 2–8 °C for up to 12 h before transferring the sample to the inactivation buffer. In the above study, nasopharyngeal swabs were collected in UTM/VTM, stored frozen, thawed, eluted in sample inactivation buffer and tested with LIAISON^®^ SARS-CoV-2 Ag according to the test procedure. In a further statement, the manufacturer recommends that each laboratory should investigate the used pre-analytical methods (storage stability) to determine the validity.

The Elecsys SARS-CoV-2 Antigen (“ECLIA”) assay from Roche Diagnostics GmbH (Mannheim, Germany) uses the antibody sandwich principle (monoclonal anti‑SARS‑CoV‑2 antibodies (mouse and rabbit)) in an electrochemiluminescence immunoassay (ECLIA) to detect SARS-CoV-2 nucleocapsid protein in nasopharyngeal and oropharyngeal swab samples from patients with signs and symptoms suggestive of COVID‑19, or known or suspected exposure to SARS‑CoV‑2 [29]. The manufacturer has described the sample stability when using three different liquid transport media in the package insert. The assay is intended for use on the cobas e411, e601 and e602 analyzers. A result of COI ≥ 1.0 can be interpreted as reactive for SARS‑CoV‑2 Ag. The manufacturer describes the clinical performance data of the test based on three cohorts and gives different sensitivities and specificities for symptomatic/asymptomatic patients with different disease duration and viral load. Values between 65.5 and 100% are given for sensitivity, and between 99.8 and 100% for specificity [29].

The SARS-CoV-2 Antigen ELISA ("ELISA") from Euroimmun Medizinische Labordiagnostika AG (Lübeck, Germany) is a semi-quantitative enzyme-linked immunosorbent assay (ELISA) for the in vitro detection of SARS-CoV-2 nucleocapsid from nasopharyngeal swabs [30]. The reaction tubes are coated with a monoclonal anti-SARS-CoV-2 nucleocapsid antibody. For interpretation of results, ratios ≥ 0.60 results are considered positive. Clinical performance was determined by the manufacturer using 98 nasopharyngeal swabs, with PCR-positive samples all from symptomatic patients < 10 days after symptom onset. A sensitivity of 93.6% and a specificity of 100% are reported [30].

### Quantitative viral load determination

The following PCR assays were used for quantification in the accredited routine diagnostics laboratory of the Max von Pettenkofer Institute [24]: the nucleocapsid (N1) reaction (Center for Disease Control (CDC) protocol [31], the nucleocapsid amplification (Seegene Allplex 2019-nCoV Assay), the Roche Cobas SARS-CoV-2 nucleocapsid reaction or the Xpert Xpress SARS-CoV-2/Flu/RSV run on the GeneXpert System. For nucleic acid extraction, the Maxwell RSC Viral Total Nucleic Acid Purification Kit was used with the Maxwell RSC-48 device (Promega GmbH, Fitchburg, USA). Quantification was based on two reference samples from INSTAND e.V. [32] with 10^6^ and 10^7^ RNA copies per ml and reference patient sample-based dilution series. The reference and dilution samples were tested in duplicate with the respective instruments and by different methods.

Standard curves, specific to each instrument and method, were generated based on the results of the analyses and calculated using factors for slope and y-intercept, natural logarithmic function equations and by calibrating the second standard dilution on the INSTAND reference material [33]. For the CDC protocol, Seegene Allplex Assay, Roche Cobas and GeneXpert System, the following formulas were used, respectively: *y* = − 146ln(*x*) + 46,721; *y* = − 1,48ln(*x*) + 45,118; *y* = − 1,401ln(*x*) + 44,576; *y* = − 1,5ln(*x*) + 45,904. In general, the calculations for quantification do not take into account variability between separate PCR runs. However, since this variability applies to all study groups, they do not affect the interpretation of the results in this study.

### Analysis of SARS-CoV-2 whole-genome sequencing

Amplicon pools covering the SARS-CoV-2 genome were prepared according to the ARTIC network nCoV-2019 sequencing protocol v2 and analyzed utilizing the Artic bioinformatics protocol, in principle as reported [34]. The consensus sequences and associated sample metadata were uploaded to the GISAID repository.

### Propagation of SARS-CoV-2 from primary patient material

Caco-2 cells (American Type Culture Collection, ATCC, Virginia, USA) in virus isolation medium (Dulbecco's modified Eagle's medium (Gibco, ThermoFisher) containing 2% fetal bovine serum (Sigma-Aldrich), 100 U/ml penicillin–streptomycin (Sigma-Aldrich), 1 × non-essential amino acids (Gibco, ThermoFisher), 0.5 µg/ml gentamicin (Ratiopharm), and 0.25 µg/ml amphotericin B (Gibco, ThermoFisher)) were challenged for 2 h with a clinical isolate (GISAID EPI ISL: 466888) previously obtained from a nasopharyngeal swab of a COVID-19 patient. Subsequently, virus isolation medium was replaced with culture medium, and 3 days post-infection the supernatant was collected and passaged onto Vero-E6 cells (American Type Culture Collection, ATCC, Virginia, USA). After three additional days, cell culture supernatants were harvested and stored at − 80 °C. Further propagation of virus was performed in the expansion medium (Dulbecco's modified Eagle's medium containing 5% fetal bovine serum (Sigma-Aldrich), 100 U/ml penicillin–streptomycin (Sigma-Aldrich) and 1 × non-essential amino acids (Gibco, ThermoFisher)). Alpha VOC (B.1.1.7; GISAID EPI ISL: 2094739) and Beta (B.1.351; GISAID EPI ISL: 1752394) were initially grown on CaCo-2 or Vero-E6 cells and then expanded on Vero-E6 cells. Virus stocks were characterized by real-time RT-PCR as reported previously [24].

### Statistical analyses

Statistical analysis was performed in R version 4.1.0, using the pROC package to perform receiver operator characteristic curve (ROC curve) analysis [35]. Binomial confidence intervals for sensitivities and specificities were computed using the Wilson score interval. To further analyze analytical sensitivities, we used logistic regression, with viral loads as independent and test outcomes as the dependent variable, yielding detection probabilities for each viral load level.

## Results

### Specificities of quantitative, automated SARS-CoV-2 antigen tests range between 97.0 and 99.7%

The specificity of four quantitative, automated SARS-CoV-2 Ag tests was compared to the qualitative POCT from Roche Diagnostics. Samples taken from nasopharynx (*n* = 118), oropharynx (*n* = 174) or unrecorded sampling site in the upper respiratory tract (*n* = 11) from 303 SARS-CoV-2 PCR-negative hospitalized adults were analyzed (Table 1). The specificity of the quantitative SARS-CoV-2 Ag tests ranged from 97.03% for CLEIA, over 97.69% for ECLIA to 99.67% for CLIA and ELISA, while RAT showed a specificity of 100% in this sample collection, higher than previously reported [3].

**Table 1:** Determination of assay specificities for four quantitative, laboratory-based Ag tests and one qualitative POCT SARS-CoV-2 Ag test using SARS-CoV-2 PCR-negative respiratory swabs from adults

Assay	Specificity (%)	95% CI (%)	False positive/total
CLEIA	97.03	94.45–98.43	9/303
CLIA	99.67	98.15–99.98	1/303
ELISA	99.67	98.15–99.98	1/303
ECLIA	97.69	95.31–98.88	7/303
RAT	100.00	98.75–100.00	0/303

Binomial confidence intervals were computed using the Wilson score interval

### Analytical performance of quantitative, automated SARS-CoV-2 antigen tests

**Fig. 1:**
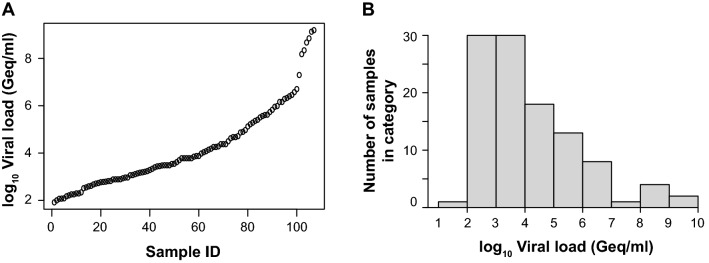
SARS-CoV-2 viral load distribution of respiratory samples included in the study. **a** Shown is the log10 viral load (Geq/ml) of all 107 SARS-CoV-2-positive patient samples, sorted by ascending magnitude from left to right. Each dot indicates one patient and the sample’s ID is indicated. **b** Depicted is the histogram of the viral load distribution by categorization of samples into defined log10 viral load value ranges. Each bar indicates the number of samples in the respective viral load range

To evaluate the analytical performance of the quantitative SARS-CoV-2 Ag tests, we included swabs taken by health-care professionals from the nasopharynx (*n* = 83), oropharynx (*n* = 19) or unrecorded sampling sites in the upper respiratory tract (*n* = 5) from 107 SARS-CoV-2 PCR-positive hospitalized adults with viral loads ranging between 83 and 1,548,572,803 Geq/ml (median: 6,045 Geq/ml; Fig. 1).

**Fig. 2:**
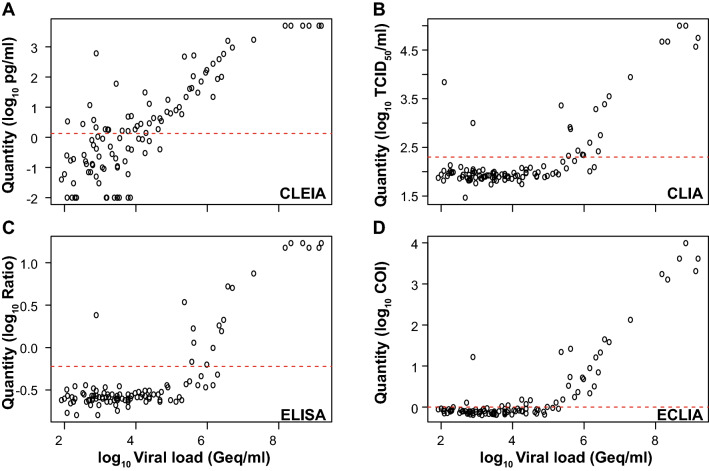
Analytical sensitivity of PCR-positive SARS-CoV-2 patient samples for quantitative SARS-CoV-2 Ag tests. **a** CLEIA from Fujirebio, **b** CLIA from Diasorin, **c** ELISA from Euroimmun and **d** ECLIA from Roche Diagnostics. The log_10_ of quantified samples were plotted against the log_10_ of the calculated viral loads. The horizontal dotted red line indicates the cutoff suggested by the manufacturer

The analytical performance of the four quantitative SARS-CoV-2 Ag tests is depicted in Fig. 2. Here, the cutoffs for positive and negative scoring were set according to the manufacturers’ recommendations. Accordingly, 56 PCR-positive SARS-CoV-2 patients were tested true positive with CLEIA, corresponding to a sensitivity of 52.34% (Fig. 2a, Table 2).

**Table 2:** Determination of assay sensitivities for four quantitative, laboratory-based Ag tests and one qualitative POCT SARS-CoV-2 Ag test for SARS-CoV-2 PCR-positive respiratory swabs from adults

Assay	Sensitivity (%)	95% CI (%)	True positive/total
CLEIA	52.34	42.96–61.55	56/107
CLIA	19.63	13.21–28.15	21/107
ELISA	17.76	11.57–26.08	19/107
ECLIA	31.42	23.34–40.83	33/105
RAT	23.58	16.52–32.50	25/106

Binomial confidence intervals were computed using the Wilson score interval

Only 21 PCR-positive swabs were tested true positive with CLIA, reflecting a sensitivity of 19.63% (Fig. 2b, Table 2). The ELISA showed a comparable, low sensitivity of 17.76% (Fig. 2c, Table 2). In the ECLIA, 33 PCR-positive COVID-19 patient samples were true positive corresponding to a sensitivity of 31.42% (Fig. 2d, Table 2). In comparison, the RAT scored 25 PCR-positive SARS-CoV-2 patients true positive, reflecting a sensitivity of 23.58% (Table 2).

**Fig. 3:**
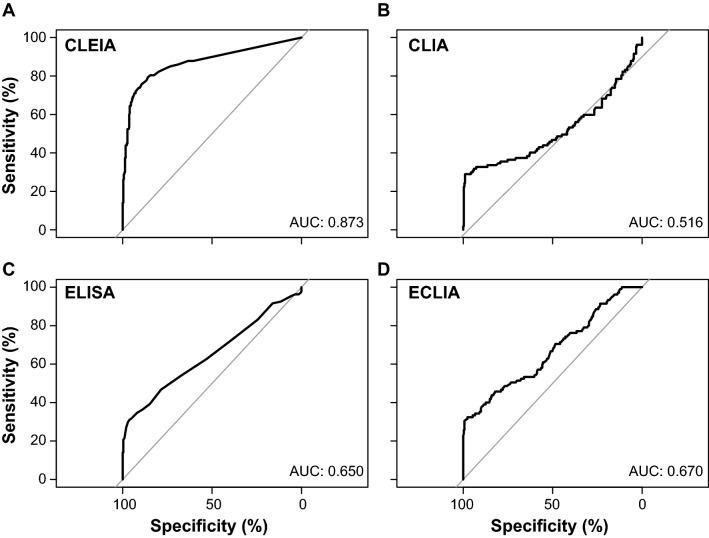
ROC analyses for quantitative SARS-CoV-2 Ag tests with **a** CLEIA, **b** CLIA, **c** ELISA and **d** ECLIA. The respective AUCs are depicted

Next, we analyzed the receiver-operating characteristics (ROCs) to evaluate the overall performance of the quantitative SARS-CoV-2 Ag tests (Fig. 3). The calculated area under the curves (AUCs) indicated that CLEIA performed best with an AUC of 0.873. Second among the four automated and quantitative SARS-CoV-2 Ag tests was ECLIA with an AUC of 0.670. In comparison, ELISA and CLIA had an AUC of 0.650 and 0.516, respectively.

Based on these ROC analyses, we then determined the Youden’s index, which is a measure of a diagnostic test's ability to balance sensitivity and specificity. The maximal Youden’s index of 0.6525 reached an optimal cutoff of 0.095 for CLEIA, yielding a sensitivity of 79% and a corresponding specificity of 86% (Table 3). Of note, the cutoff with the calculated Youden’s index was significantly lower than that recommended by the manufacturer [27]. Aiming for a minimal sensitivity of 80%, recommended by the WHO for SARS-CoV-2 rapid Ag tests, would lower the specificity of CLEIA to 85% only. Conversely, a specificity of 97% would decrease the corresponding sensitivity to 52%. For CLIA, the sensitivity and specificity were 27% and 99%, respectively, with a maximal Youden’s index of 0.2611 and a cutoff of 115.92. As a consequence, aiming at a sensitivity of 80% would lower the specificity to only 11%, while a specificity of 97% would yield a sensitivity of 27%. The ELISA showed an optimal cutoff of 0.325 with a Youden’s index of 0.2721 and a sensitivity and specificity of 31% and 96%, respectively. Here, a sensitivity of 80% would drastically reduce the specificity to 29%, yet a target specificity of 97% would only marginally decrease the sensitivity to 30%. Only the cutoff based on the calculated Youden’s index (0.3007) for ECLIA was very close to the manufacturer’s recommendation and reached a sensitivity and specificity of 33% and 98%, respectively. The specificity of this assay would have to be lowered to 30% to reach a sensitivity of 80%. Conversely, a target specificity of 97% would only marginally decrease the sensitivity to 32%.

**Table 3:** Calculation of cutoffs based on the Youden’s indices for four quantitative, laboratory-based SARS-CoV-2 Ag tests

Assay	Sensitivity (%)	Specificity (%)	Max. Youden's index	Cutoff	Manufacturer's recommendation
CLEIA	79.44	85.81	0.6525	0.095	≥ 1.34
CLIA	27.10	99.01	0.2611	115.915	≥ 200
ELISA	30.84	96.37	0.2721	0.325	≥ 0.60
ECLIA	32.38	97.69	0.3007	0.9915	≥ 1.00

**Fig. 4:**
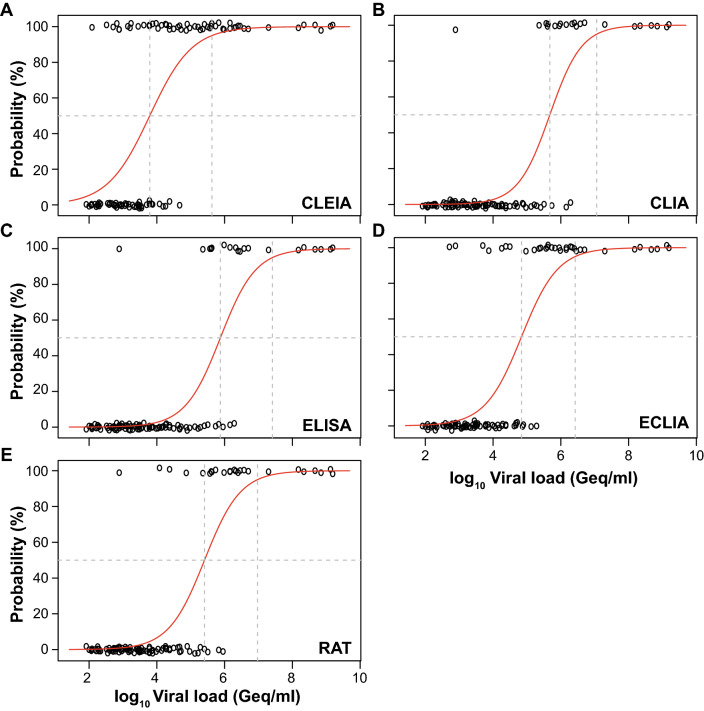
Limit of detection analyses of PCR-positive SARS-CoV-2 patient samples for quantitative SARS-CoV-2 Ag tests: **a** CLEIA, **b** CLIA, **c** ELISA, **d** ECLIA, and **e** the POCT RAT. The log10 viral load of quantified samples on the *x*-axis was plotted against a positive (+ 1) or negative (0) test outcome on the *y*-axis. For readability of the figure, slight normal jitter was added to the *y*-values. Red curves show logistic regressions of the viral load on the test outcome; horizontal dashed line shows 50% detection probability, whereas vertical dashed lines indicate log viral loads at which 50% (LoD_50_) and 95% (LoD_95_), respectively, of the samples are expected positive based on the regression results

We further assessed the analytical sensitivity of the quantitative and qualitative SARS-CoV-2 Ag tests (Fig. 4) by calculating the 50% and 95% limits of detection (LoD) based on a logistic regression model as recently reported [1]. The virus concentrations at which 50% and 95% detection rates were achieved with CLEIA were 6,181 and 422,689 Geq/ml, respectively. In comparison, LoD_50_ and LoD_95_ values for CLIA were 473,279 and 11,452,782 Geq/ml, i.e. 77 and 27 times higher, respectively. The performance of the ELISA was even worse with 121 and 61 times higher LoD_50_ and LoD_95_ values, respectively, corresponding to 749,792 and 25,711,669 Geq/ml. The LoD_50_ and LoD_95_ for ECLIA were only 11- and 6-times lower than those for the CLEIA, corresponding to 69,002 and 2,654,696 Geq/ml, respectively. In comparison to the quantitative SARS-CoV-2 Ag tests, the LoD_50_ and LoD_95_ values of RAT yielded 255,537 and 9,324,079 Geq/ml, respectively, i.e. 41 and 22 times higher relative to CLEIA.

### Quantitative, automated SARS-CoV-2 antigen tests are able to detect VOCs Alpha and Beta

**Fig. 5:**
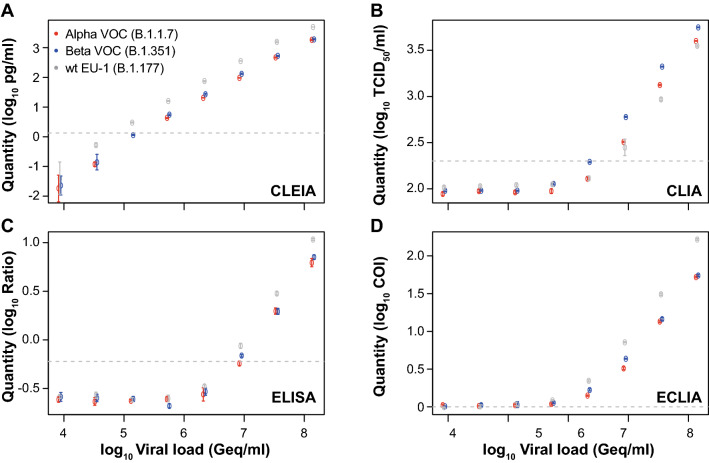
Quantification of VOCs using the quantitative SARS-CoV-2 Ag tests: **a** CLEIA, **b** CLIA, **c** ELISA and **d** ECLIA. The quantified samples were plotted against the calculated viral loads

Given the increasing genetic diversification of SARS-CoV-2 and the emergence of VOCs, which carry mutations not only in spike but also in nucleocapsid, we assessed whether these quantitative SARS-CoV-2 Ag tests are also able to detect VOCs Alpha and Beta. First, we evaluated the detection of clinical SARS-CoV-2 isolates that had been expanded in tissue culture and confirmed as a VOC by whole-genome sequencing [34]. As shown in Fig. 5, both VOCs Alpha and Beta were detected by all quantitative Ag tests. While CLEIA was able to detect viral loads of an isolate from 2020 (EU1, B.1.177) as well as VOCs Alpha and Beta down to 1 × 10^5^ Geq/ml, CLIA and ELISA were only able to reliably detect viral loads up to 8 × 10^6^ Geq/ml. This was in a range similar to RAT, which was tested in parallel (data not shown). The ECLIA was slightly more sensitive and able to detect 5 × 10^5^ Geq/ml for Alpha and 2 × 10^6^ Geq/ml for Beta.

Second, the genotype of SARS-CoV-2 in respiratory swabs taken from 79 COVID-19 patients was determined by whole-genome sequencing. Since February of 2021 fell into the late phase of the second pandemic wave in Germany, 14% of our respiratory samples were already Alpha positive. Of note, the Alpha-positive patients’ median viral load was 1,929,014 Geq/ml, i.e. 320 times higher than the median of non-VOC SARS-CoV-2-positive respiratory samples from COVID-19 patients included in the study. Among the 19 COVID-19 patients with primary diagnosis, 4 were infected with VOC Alpha (21%). Non-VOC SARS-CoV-2 was detected in seven patients (36.8%) and for eight patients no genotypic information was available. Although the subgroup of patients with primary diagnosis was rather small, Alpha-infected patients had also here a median viral load of 12,424,703 Geq/ml compared to 9,528 Geq/ml in non-VOC-positive patients.

## Discussion

In the current study, we evaluated the performance of four commercial, automated SARS-CoV-2 Ag tests that were launched on the European market in early 2021 for the quantitative detection of SARS-CoV-2 in respiratory samples from COVID-19 patients hospitalized at the LMU Klinikum and three LMU München teaching hospitals as well as on cell culture-expanded clinical isolates, including VOCs. We assessed the sensitivity and specificity as well as method-based test characteristics of these four laboratory-based Ag assays and also compared them to a widely used POCT.

The minimum requirements of performance recommended by international organizations for rapid SARS-CoV-2 Ag tests (sensitivity ≥ 80%, specificity > 97%) are certainly not simply transferable to automated Ag assays. Since this study was conducted at a time during the pandemic when the number of newly infected individuals in Germany was declining at the end of the second pandemic wave, it was difficult to include primarily fresh respiratory samples from patients at the time of initial diagnosis and presumably high viral load. Therefore, the low clinical sensitivities, which only have relevance in the specific cohort investigated, are not discussed further. The analytical sensitivity (95% detection limit) of the four automated SARS-CoV-2 Ag tests examined ranged from 420,000 to 25,000,000 Geq/ml. In comparison, the detection limit of the Roche Rapid Ag Test was 9,300,000 Geq/ml, which is also in good agreement with a recent report [1].

This indicates that automated Ag testing does not necessarily go along with an increased sensitivity. However, it is evident that test indication and overall usefulness of automated Ag tests cannot be generalized. Rather, cutoffs must be individually adapted to and evaluated for the specific diagnostic focus. The ROC analyses in this study demonstrate impressively that lowering the cutoff for either specificity or sensitivity is not suitable for every test. In the case of CLIA, ELISA and ECLIA, it is evident that cutoffs have already been set by the manufacturers just above the lower asymptote of negative results. Only CLEIA shows a wide dynamic range that allows marked variations. For this assay, the manufacturer also explicitly recommends adjusting the cutoff to the requirements of the specific laboratory performing the assay. Recently, Häuser et al*.* performed similar analyses on the automated CLIA [12]. In their study cohort of more than 130 COVID-19 patients hospitalized in the period December 9, 2020 to January 29, 2021, the overall analytical sensitivity was 40.2% at a specificity of 100%, while lowering the assay cutoff from 200 to 100 arbitrary units (AU) per mL increased the sensitivity to 49.7% while decreasing the specificity to 98.3%.Possible areas of application of automated Ag tests are the screening of asymptomatic patient groups with regard to possible infectivity at the time of testing during a phase of increased incidence or testing for de-isolation of known PCR-positive patients in the hospital setting in addition to local symptoms-dependent hygiene concepts.

The specificity of an Ag test determines the number of false-positive results depending on the pre-test probability. Independent studies and meta-analyses [2] now show that high specificity can be achieved with SARS-CoV-2 rapid Ag tests [2, 3]. In our current analysis, the RAT used confirms this observation. Independent testing and summaries of automated Ag test specificities are also important, as substantial variability can occur due to individually definable cutoff values for tests that have a quantitative or semi-quantitative output measure. In our current study, all Ag tests investigated were able to stay above the limit required by the WHO for the specificity of SARS-CoV-2 rapid Ag tests of more than 97%. To our knowledge, there is no corresponding recommendation for automated laboratory Ag tests from any official institution. Laboratory operators should responsibly and proactively plan the use of automated Ag tests depending on local incidence and circulating variants, the latter potentially also affecting assay performance.

Undoubtedly, any positive Ag test, POCT or laboratory based, has to be confirmed by PCR. Specificities of around 97%, as found in our current study for the CLEIA and ECLIA, can lead to significant rates of false-positive results and corresponding burden on laboratory workflows due to a need for PCR retesting with associated delays in the time to result.

Recent analyses showed that individuals infected with VOC Alpha had a viral load at primary diagnosis of one order of magnitude higher than individuals infected with a non-VOC [36–41]. We were able to observe this trend between these two patient groups, despite the small number of COVID-19 cases. As a consequence, Alpha VOC-infected patients may be more likely to be recognized by an Ag test early during the course of infection. Unfortunately, we were unable to demonstrate such an effect, likely due to the small number of primary diagnoses. In this context, however, it is more important to investigate whether different virus variants—especially VOCs—can be detected by automated Ag tests with comparable sensitivity. Changes in the virus genome can lead to non-synonymous amino acid exchanges. In the spike protein, for example, the polymorphisms K417N and E484K in VOC Beta can reduce the efficacy of neutralizing antibodies. The nucleocapsid gene harbors a mutational hotspot in the region around amino acid positions 202–205, which is probably an important phosphorylation site for packaging. While these SNPs are not VOC specific, almost all current SARS-CoV-2 lineages carry mutations in this area of the genome compared to the Wuhan reference sequence. In addition to these four amino acid positions, there are five other amino acids in nucleocapsid that can differ between VOCs and the early pandemic isolates (D3, D63, P80, G215, S235 and D377). Mutations in the nucleocapsid of the major VOCs and the wild-type virus used in this study are shown in Table 4 [23].

**Table 4:** Summary of known amino acid mutations with > 75% prevalence in VOCs Alpha, Beta, Gamma and Delta and EU1/B.1.177 from March 2020 used in this study with non-synonymous amino acid substitutions at the indicated amino acid positions in the nucleocapsid of SARS-CoV-2

Amino acid position	D3	D63	P80	R203	G204	T205	G215	A220	S235	D377
EU1								V		
Alpha	L			K	R				F	
Beta						I				
Gamma			R	K	R					
Delta		G		M			C			Y

Source: https://outbreak.info/situation-reports

Four of the five assays reported in this study are based on monoclonal antibodies that bind to the viral nucleocapsid protein in the immunoassay. Only CLIA uses polyclonal antibodies for Ag detection and Fujirebio states for the CLEIA that they use a mixture of different monoclonal antibodies. Although the stage of infection and pre-analytical conditions may have the greatest impact on a false-negative Ag test result, it is essential to also investigate failures in the binding ability of the antibodies used in these assays due to VOC-specific mutations. An immunoassay that includes polyclonal antibodies certainly has a conceptual advantage of reliably detecting multiple epitopes on the nucleocapsid protein even if mutations occur at a single site, whereas the use of a monoclonal antibody in the assay design is more vulnerable to drastic loss of binding and assay failure [42]. Since different manufacturers are likely to have the diagnostic antibodies bind in different domains of the nucleocapsid protein, this survey must be repeated for each test on the market. It can be assumed that Ag test antibodies react to more conserved domains of the nucleocapsid, but so far this information has not been disclosed by the manufacturers. In our experiments with supernatants from cell culture of primary patient clinical isolates of Alpha and Beta, we did not observe a marked drop in test sensitivity. This result is in line with the findings of a recent publication in which no difference was found between cell culture supernatants of Alpha and Beta VOC isolates and non-VOC isolates for detection rates in RATs [43].

In summary, automated assays for the detection of SARS-CoV-2 nucleocapsid protein from respiratory samples significantly differ in their assay dynamics, with marked differences in analytical sensitivity. However, sensitivity is not consistently higher in automated, laboratory-based assays compared to a widely used RAT and no marked difference in detecting a SARS-CoV-2 isolate from early 2020 relative to VOC Alpha and Beta could be established. Nucleic acid-based testing remains the gold standard for high-performance SARS-CoV-2 detection and quantification in a laboratory setting.

## Data Availability

Not applicable.
